# Predictors of the Short‐Term Outcomes of Guillain–Barré Syndrome: Exploring Electrodiagnostic and Clinical Features

**DOI:** 10.1002/brb3.70257

**Published:** 2025-01-20

**Authors:** Yi‐Hsiang Chen, Chia‐Lun Wu, Wei‐Chieh Weng, Yi‐Chia Wei

**Affiliations:** ^1^ Department of Neurology Chang Gung Memorial Hospital Keelung Taiwan; ^2^ College of Medicine Chang Gung University Taoyuan Taiwan

**Keywords:** electrodiagnostic, electromyography, Guillain–Barre syndrome, nerve conduction studies, outcome prediction

## Abstract

**Background and Objectives:**

Guillain–Barré syndrome (GBS), an acute inflammatory disorder of the peripheral nervous system, is characterized by muscle weakness and paralysis. Prompt identification of patients at a high risk of poor outcomes is crucial for timely intervention. In this study, we combined clinical data with nerve conduction study and electromyography data to identify the predictors of GBS outcomes.

**Methods:**

We retrospectively analyzed the data of patients with GBS who had received treatment at Chang Gung Memorial Hospital, Taiwan, between 1998 and 2022. Comprehensive clinical and electrophysiological data were collected. Statistical analyses were performed to identify the predictors of poor outcomes. The patients were stratified into two groups by their scores on the GBS Disability Scale: good (score ≤ 2) and poor (score > 2) outcome groups.

**Results:**

The study finally included 24 GBS patients (mean age: 53.0 ± 20.9 years; female‐to‐male ratio: 2.3; good outcome group: 13; poor outcome group: 11). Compared with the good outcome group, the poor outcome group was old (43.0 ± 20.4 vs. 64.0 ± 15.7, *p =* 0.011), had a short time‐to‐treatment period (12.9 ± 7.8 vs. 6.5 ± 5.4 days, *p =* 0.033), exhibited more prevalent mechanical ventilation use (0 vs. 36.4%, *p =* 0.017), and had a prolonged hospitalization duration (14.7 ± 10.2 vs. 53.1 ± 20.0 days, *p* < 0.001). Poor outcomes were associated with low compound muscle action potential (CMAP), slow motor nerve conduction velocity (MNCV), abnormal F‐wave latency, and more conduction block and temporal dispersion. In the subgroup of acute inflammatory demyelinating polyradiculoneuropathy (AIDP), there were 19 patients, out of which 10 had good outcomes, while nine had poor outcomes. The clinical features that differentiate between good and poor outcomes in the AIDP subgroup were similar to those observed in all GBS patients. Notably, the motor conduction features, including distal and proximal CMAP and MNCV of the median and tibial nerves (all *p* < 0.05), were particularly important electrodiagnostic features of outcome discrimination in the AIDP subgroup.

**Discussion:**

Combining clinical data with nerve conduction study and electromyography data can assist in predicting outcomes of both GBS patients and the AIDP subgroup. Poor outcomes are associated with older age, a more abrupt onset pattern, low CMAP, and slow nerve conduction, and abnormal tibial F responses can predict poor outcomes. Early identification of high‐risk patients facilitates tailored interventions. This highlights the importance of combining clinical and electrophysiological data in GBS management.

## Graphical abstract

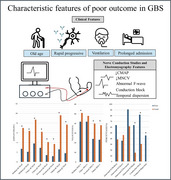

## 1 Introduction

Guillain–Barré syndrome (GBS) is a rare but critical neurological disorder characterized by sudden‐onset muscle weakness and, in severe cases, paralysis. The annual global incidence of GBS is approximately one to two per 100,000 person‐years (Lee et al. 2016; Leonhard et al. 2019; Rajabally and Uncini 2012; Shangab and Al Kaylani 2020). GBS is an acute inflammatory disease of the peripheral nervous system and is proposed to be mediated by antiganglioside antibodies against the paranodal region (Kieseier et al. 2018) or Schwann cells (Pandey and Mudgal 2022). Microbiomes infections could be a provoking factor of GBS (van den Berg et al. 2014; Wakerley and Yuki 2013; Ziganshin et al. 2016) and induce molecular mimicry (Kaida, Ariga, and Yu 2009; Wakerley and Yuki 2013), and subsequent complement activation (Wanschitz et al. 2003) and myelin (Koski 1990) and axonal injuries (He et al. 2015).

The symptoms of GBS include symmetrical weakness, areflexia. GBS may show increased protein levels in the cerebrospinal fluid study (Leonhard et al. 2019). These symptoms can rapidly intensify, necessitating immediate medical intervention. GBS‐induced damage to the autonomic nervous system and respiratory muscles is a potentially life‐threatening condition (Shang et al. 2021). Aggressive and prompt immunotherapies, such as plasmapheresis and intravenous immunoglobulin treatment, can effectively reduce the severity and duration of symptoms and eliminate the sequelae of GBS (Davidson et al. 2017; Querol and Lleixa 2021; Shang et al. 2021; Wang et al. 2017). Therefore, early identification of patients at high risk of severe disease and poor outcomes is crucial for the effective management of GBS (Park and Kim 2016; Wakerley, Uncini, and Yuki 2014; Walgaard et al. 2011; Wang et al. 2017).

A nerve conduction study (NCS) and electromyography are basic electrophysiological tests used to assess the peripheral nervous system. These tests help detect nerve injury in patients with GBS (Alexander et al. 2011), elucidate mechanisms underlying GBS‐induced nerve injury, identify electrophysiological deficits in these patients, and differentiate between GBS subtypes. Demyelination is the most common nerve injury in GBS (Yoon et al. 2020), and NCS and electromyography can detect different segments of myelin injury in GBS. For example, F‐wave latency of involved nerves is prolonged or absent, and the H reflex is missing in proximal demyelination. Motor nerve conduction velocity (MNCV) is decreased, and conduction blocks and temporal dispersion may be observed in segmental demyelination. Distal demyelination is characterized by prolonged distal motor latency (DML) and reduced distal compound muscle action potential (CMAP). Besides, *sural nerve sparing* in sensory nerve conduction studies is considered a feature of demyelinating disease and presents in early course of any GBS subtype (Al‐Shekhlee, Robinson, and Katirji 2007). In contrast, axonal injuries, whether primary or secondary to demyelination, lead to a simultaneous reduction in CMAP at both proximal and distal sites, with or without a reduction in sensory nerve action potential. The presence of fibrillation, positive sharp waves, and reduced recruitment on electromyography is indicative of acute denervation. Therefore, NCS and electromyography can be used to diagnose GBS, classify its subtypes, and obtain prognostic insights. These electrodiagnostic features are potentially important predictors of GBS outcomes.

In this study, we explored the use of NCS and electromyography data along with clinical data for identifying the predictors of short‐term GBS outcomes. Our findings may facilitate the early identification of patients at a high risk of poor prognosis, thereby enabling health‐care professionals to predict and manage clinical outcomes in these patients in time. By examining diverse parameters, such as demographic characteristics, temporal treatment features, and clinical presentations, and using rigorously validated GBS severity and outcome assessment scales, we provide comprehensive insights into GBS. Furthermore, we combined electrodiagnostic data with clinical data to identify high‐risk patients. This integrated approach can improve the quality of care for these patients by guiding life‐saving interventions and optimizing overall clinical outcomes.

## 2 Methods

### 2.1 Patient Enrollment

This retrospective study comprised patients who had visited the Department of Neurology at Chang Gung Memorial Hospital, Keelung, Taiwan, between 1998 and 2022. We included patients who had developed the initial symptoms of GBS and had undergone complete electrodiagnostic tests. We excluded patients whose electrophysiological results did not align with GBS features and those who did not receive a final diagnosis of GBS. We also excluded patients whose electrodiagnostic testing was unavailable or deferred.

We perform an NCS study as soon as possible after admission and suspected GBS. The examination times of patients were in 3 weeks. The clinical diagnostic criteria for GBS (Asbury and Cornblath 1990; Leonhard et al. 2019) include rapidly progressing bilateral limb weakness and sensory deficits, hyporeflexia or areflexia, facial or bulbar palsy, ophthalmoplegia, and ataxia. These symptoms must develop within 4 weeks to fit the diagnostic criteria. For inclusion in our study, patients had to meet a specific electrodiagnostic criterion in at least 2 nerves. An NCS was conducted to measure distal CMAP, proximal CMAP, MNCV, DML, temporal dispersion, conduction block, and F‐wave latency (Lee et al. 2016). Abnormal electrodiagnostic criteria were as follows:

1. CMAP values of <4, <6, <2, and <4 mV for the median, ulnar, peroneal, and tibial nerves, respectively.
2. MNCV of <50 and <40 m/s for the median and ulnar nerves and the peroneal and tibial nerves, respectively.
3. DML of >4.5, 3.5, >6.5, and >6 ms for the median, ulnar, peroneal, and tibial nerves, respectively.
4. Temporal dispersion is the “relative desynchronization of components of a CMAP due to different rates of conduction of each synchronously evoked component from the stimulation point to the recording electrode” in the American Association of Electrodiagnostic Medicine (AAEM) glossary of terms (Phillips et al. 2002). The definition of temporal dispersion was indicated by a >20% increase in the duration of proximal CMAP with a reduction in proximal CMAP amplitude (compared with distal CMAP amplitude) (Mallik and Weir 2005).
5. Conduction block is the “failure of an action potential to propagate past a particular point in the nervous system whereas conduction is possible below the point of the block” (Phillips et al. 2002). It is defined by >20% reduction in CMAP area or amplitude during proximal versus distal nerve stimulation, except in the tibial nerve, for which >50% reduction must be observed in proximal CMAP area or amplitude (Mallik and Weir 2005).
6. F wave is “an action potential evoked intermittently from a muscle by a supramaximal electric stimulus to the nerve due to antidromic activation of motor neurons” (Phillips et al. 2002). Abnormal F‐wave latency is defined as a prolonged latency compared to the estimated latency. For the median and ulnar nerves, F‐wave latency over 5 ms is considered prolonged, while for the peroneal and tibial nerves, latency over 7 ms is considered prolonged. An absence of F response is also regarded as an abnormal F response.

### 2.2 Clinical Data Collection

We collected clinical data on patient age, sex, duration of hospitalization, time of symptom onset, timing of NCS and electromyography tests, initial treatments received, history of infections before symptom onset, clinical presentations (e.g., facial and bulbar palsy), history of mechanical ventilation, and treatment choices.

### 2.3 NCS and Electromyography Tests

NCS and electromyography tests were performed on the basis of the initial suspicion of GBS. The median, ulnar, peroneal, and tibial nerves were tested (at least one of each nerve type). An NCS was performed to measure distal CAMP, proximal CMAP, MNCV, sensory nerve conduction velocity, F waves, and H reflexes. Needle electromyography was performed after obtaining informed consent. Although a second set of NCS and electromyography tests was not mandatory for all patients, these tests were performed in cases of disease progression or when a follow‐up electrodiagnostic evaluation was necessary after the first course of plasmapheresis or intravenous immunoglobulin treatment.

### 2.4 GBS Severity Rating Scales

#### 2.4.1 Medical Research Council Scale

The Medical Research Council (MRC) scale is used to assess patients with GBS experiencing secondary clinical deterioration after treatment. This tool measures muscle strength across six bilateral muscle groups: arm abduction, forearm flexion, wrist extension, leg flexion, knee extension, and foot dorsiflexion. The movement of each muscle group is rated on a scale with end points ranging from 0 to 5. The MRS sum score ranges from 0 to 60 (Kleyweg et al. 1991). Furthermore, the MRS sum score is incorporated into the modified Erasmus GBS Outcome Score (mEGOS) (Walgaard et al. 2011) and the Erasmus GBS Respiratory Insufficiency Score (EGRIS) (Walgaard et al. 2010).

#### 2.4.2 GBS Disability Scale

The GBS Disability Scale was developed in a trial (1987) investigating the benefits of prednisolone for patients with acute polyneuropathy. This scale assesses the long‐term outcomes of GBS. Responses are rated on a scale with end points ranging from 0 to 6, with intermediate values representing varying levels of disability. Scores of 0, 1, 2, 3, 4, 5, and 6 indicate health, minor symptoms but ability to run, ability to walk ≥5 m without assistance but inability to run, requirement of assistance to walk ≥5 m across an open space, being bedridden or chair‐bound, requirement for assisted ventilation for at least part of the day, and death, respectively (Hughes et al. 1978).

### 2.5 GBS Outcome Measures

#### 2.5.1 Modified Erasmus GBS Outcome Score

The Erasmus GBS Outcome Score is a scoring system developed to predict the 6‐month outcomes of GBS on the basis of patient's clinical characteristics during the acute phase of the disease (van Koningsveld et al. 2007). However, this tool is typically used in the second week after admission, which introduces a delay from the initial patient visit. To address this problem, the mEGOS (Walgaard et al. 2011) was introduced to predict GBS outcomes at hospital admission, when treatment is initiated. A follow‐up mEGOS assessment is performed 1 week after admission. This score may be used in therapeutic trials (Walgaard et al. 2011). The mEGOS is useful for predicting not only 6‐month disability outcomes but also patients’ ability to walk independently or exhibit improvement in the GBS disability score by the fourth week after the initial visit.

The mEGOS assesses three components: age at onset (scores of 0, 1, and 2 for patients aged <40, 40–60, and >60 years, respectively), presence or absence of preceding diarrhea (scores of 1 and 0 indicate presence and absence, respectively), and adjustments to the MRC sum scores at hospital admission and a week after admission. The sum score at admission is converted from the original MRC score to a scale with the following points: 0, 2, 4, and 6 points indicate MRC sum scores of 51–60, 41–50, MRC 31–40, and ≤30, respectively. Regarding postadmission scores, the MRC sum score on Day 7 is adjusted to a scale with the following points: 0, 3, 6, and 9 points indicate MRC sum scores of 51–60, 41–50, 31–40, and ≤30, respectively (Walgaard et al. 2011).

### 2.6 EGRIS

The EGRIS was designed for the prediction of respiratory insufficiency in patients with GBS. The respiratory muscles of these patients may be affected by GBS; consequently, these patients may require mechanical ventilation. Requirements for mechanical ventilation and intensive unit care serve as the predictors of poor outcomes (Kumar et al. 2021). The EGRIS has three components: interval (days) between muscle weakness onset and hospital admission (score: 0–2), facial or bulbar weakness (score: 0 or 1), and MRC sum score at hospital admission (score: 0–4). The total EGRIS ranges from 0 to 7 (Green, Baker, and Subramaniam 2018). On the basis of this score, patients can be categorized into three groups with varying risks of respiratory failure: low (EGRIS: 0–2), intermediate (EGRIS: 3 or 4), and high (EGRIS: 5–7) (Walgaard et al. 2010).

### 2.7 Outcome Definitions

The patients’ postadmission 4‐week scores on the GBS Disability Scale were used to assess their clinical outcomes because most patients with GBS recover within 4 weeks of treatment (Rajabally and Uncini 2012; Walgaard et al. 2011). After a 4‐week follow‐up, we categorized the patients into the good outcome group (GBS Disability Scale score ≤ 2) or the poor outcome group (GBS Disability Scale score > 2); poor outcomes were equivalent with the inability to walk 10 m independently (van Koningsveld et al. 2007; Walgaard et al. 2011)

### 2.8 Statistical Analysis

Statistical analyses were performed using SPSS (version 20; IBM Corporation, Armonk, NY, USA). Continuous variables were analyzed using the independent *t* test and are presented in terms of the mean ± SD values. Categorical variables were analyzed using the chi‐square test and are presented in terms of the odds ratio (OR) and 95% confidence interval (CI) values. A *p* value of <0.05 indicated statistical significance.

## 3 Results

### 3.1 Patient Demographics

**FIGURE 1:**
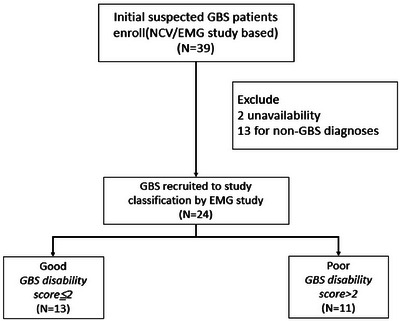
Flowchart depicting patients enrollment. Between 1998 and 2022, 39 patients with initial impression of GBS underwent comprehensive clinical, laboratory, neuroimaging, and electrodiagnostic tests. From these patients, we excluded 13 with non‐GBS diagnoses, such as chronic inflammatory demyelinating polyradiculoneuropathy (CIDP; *n* = 2), multiple myeloma (*n* = 1), paraneoplastic neuropathy (*n* = 1), multiple motor neuropathy with conduction block (MMNCB; *n* = 1), Bechet disease (*n* = 1), arsenic intoxication (*n* = 1), myelitis (*n* = 4), and compressive radiculomyelopathy (*n* = 2). Another two patients were excluded because of the unavailability of their in‐hospital medical records. Finally, 24 patients with GBS were included in this study. The patients were divided them into two groups on the basis of their scores on the GBS Disability Scale (measured in the fourth week following the initial visit): good and poor outcome groups. Patients with a score of ≤2 were included in the good outcome group, whereas those with a score of >2 were included in the poor outcome group.

This study initially included 39 patients. Among them, 13 were excluded because they had a condition other than GBS: two patients had chronic inflammatory demyelinating polyradiculoneuropathy, one had multiple myeloma, one had paraneoplastic neuropathy, one had multiple motor neuropathy with conduction block, one had Behçet's disease, one had arsenic intoxication, four had myelitis, and two had compressive radiculomyelopathy. In addition, two patients were excluded because of the unavailability of comprehensive in‐hospital data. Finally, 24 patients were included in this study (mean age 53.0 ± 20.9 years; female‐to‐male ratio 2.3). According to the outcome definitions, 13 and 11 patients were included into the good and poor outcome groups, respectively (Figure 1).

### 3.2 Between‐Group Differences in Clinical Features

Compared with the poor outcome group, the good outcome group was young (64.0 ± 15.7 vs. 43.0 ± 20.4 years, respectively; *p =* 0.011), had a less urgent onset pattern with a prolonged time‐to‐treatment period (interval between symptom onset and hospital admission; 8.1 ± 8.1 vs. 3.6 ± 6.0 days, respectively; *p =* 0.033), reduced mechanical ventilator use (36.4% vs. 0%, respectively; *p =* 0.017), and a short hospitalization duration (53.1 ± 20.0 vs. 14.7 ± 10.2 days, respectively; *p* < 0.001; Table 1).

**TABLE 1:** Clinical features of the GBS patients.

		Good (N = 13)	Poor (N = 11)	*p* value	OR (95% CI)
Demographics					
Age (years)		43.0 ± 20.4	64.0 ± 15.7	0.011^*^	
Sex male N (%)		4 (30.8)	3 (27.3)	0.851	0.8 (0.1–5.0)
Clinical features					
Preceding infection, N (%)	URI	1 (7.7)	4 (36.4)	0.085	6.9 (0.6–74.2)
	Diarrhea	1 (7.7)	2 (18.2)	0.439	2.7 (0.2–34.2)
Facial or bulbar involvement, N (%)		5 (38.5)	3 (27.3)	0.562	
Onset to hospital (days)		8.1 ± 8.1	3.6 ± 6.0	0.146	
Onset to electromyography (days)		12.7 ± 7.6	8.2 ± 7.1	0.150	
Onset to treatment (days)		12.9 ± 7.8	6.5 ± 5.4	0.033^*^	
Length of hospital stay (days)		14.7 ± 10.2	53.1 ± 20.0	<0.001^*^	
Need mechanical ventilator, N (%)		0 (0)	4 (36.4)	0.017^*^	
Treatment, N (%)	DFPP	11 (84.6)	11 (100)	0.174	
	IVIG	2 (15.4)	0 (0)	0.174	
Laboratory data					
CSF protein		113.0 ± 55.8	120.2 ± 100.5	0.834	
CSF white blood cell count		2.7 ± 2.9	2.8 ± 4.6	0.935	
GBS type					
	AIDP	10 (76.9)	9 (81.8)	0.327	
	Axonal	1 (7.7)	2 (18.2)		
	Millar–Fisher	2 (15.4)	0 (0)		
Severity of disease					
MRC sum scores at admission		53.7 ± 5.6	48.1 ± 10.3	0.128	
MRC sum scores at first week		54.4 ± 5.7	31.5 ± 15.1	<0.001^*^	
MRC sum scores at fourth week		59.5 ± 1.7	35.4 ± 14.9	<0.001^*^	
GBS disability scale at admission		1.7 ± 0.6	2.7 ± 1.6	0.059	
GBS disability scale at first week		1.5 ± 0.9	4.2 ± 0.8	<0.001^*^	
GBS disability scale at fourth week		1.2 ± 0.4	3.8 ± 0.6	<0.001^*^	
Outcome scores					
mEGOS at admission		1.4 ± 1.2	3.3 ± 2.1	0.016^*^	
mEGOS at first week		1.6 ± 1.7	7.6 ± 2.2	<0.001^*^	
EGRIS at admission		1.6 ± 1.1	2.9 ± 0.8	0.005^*^	

Abbreviations: AIDP, acute inflammatory demyelinating polyradiculoneuropathy. CSF, cerebrospinal fluid; DFPP, double filtration plasmapheresis; EGRIS, Erasmus GBS Respiratory Insufficiency Score; IVIG, intravenous immunoglobulin; mEGOS, modified Erasmus GBS Outcome Score; MRC, Medical Research Council; URI, upper respiratory tract infection. * Statistical significance at a p‐value <0.05.

### 3.3 Between‐Group Differences in Clinical Severity and Outcome Scores

The MRC sum score and GBS Disability Scale score differed significantly between the groups in Weeks 1 (54.4 ± 5.7 vs. 31.5 ± 15.1, *p* < 0.001 and 1.5 ± 0.9 vs. 4.2 ± 0.8, *p* < 0.001, respectively) and 4 (59.5 ± 1.7 vs. 35.4 ± 14.9, *p* < 0.001 and 1.2 ± 0.4 vs. 3.8 ± 0.6, *p* < 0.001, respectively). However, no significant between‐group difference was observed at admission (*p =* 0.128 and *p =* 0.059, respectively). By contrast, both the mEGOS and EGRIS were consistently lower in the good outcome group than in the poor outcome group from the time of admission (mEGOS: 1.4 ± 1.2 vs. 3.3 ± 2.1, *p =* 0.016; EGRIS: 1.6 ± 1.1 vs. 2.9 ± 0.8, *p =* 0.005; Table 1).

### 3.4 Between‐Group Differences in Electromyographic Features

The NCS revealed that distal CMAP, proximal CMAP, and MNCV for all major nerves were decreased in the poor outcome group than in the good outcome group (all *p* < 0.05; Table 2A). However, no significant between‐group difference was noted in sensory nerve conduction study (Table 2B). Furthermore, the poor outcome group exhibited more abnormalities in F‐wave latency for the tibial nerve than the good outcome group (63.6% vs. 15.4%, *p =* 0.015; Table 2C). The poor outcome group also had fewer instances of conduction block and temporal dispersion did the good outcome group (81.8% vs. 30.8%, *p =* 0.012 and 45.5% vs. 0%, *p =* 0.006, respectively; Table 2D). Moreover, the poor outcome group exhibited more features of axonal damage, as indicated by CMAP thresholds for the median, ulnar, peroneal, and tibial nerves, than the good outcome group (all *p* < 0.05; Table 2E).

**TABLE 2:** Nerve conduction studies and electromyography features of the GBS patients.

(A) Motor nerve conduction study					
	Good (*n* = 13)	Poor (*n* = 11)	*p* value		
Median DML (ms)	6.5 ± 4.8	8.5 ± 5.9	0.366		
Median dCMAP (mV)	7 ± 3.2	2.7 ± 2.2	0.001^*^		
Median pCMAP (mV)	6.4 ± 3.3	2.1 ± 1.7	0.001^*^		
Median MNCV (m/s)	50.9 ± 5	41.9 ± 9.2	0.008^*^		
Ulnar DML (ms)	3.6 ± 2.5	4.1 ± 1.6	0.610		
Ulnar dCMAP (mV)	8.7 ± 5.9	3.8 ± 2.5	0.018^*^		
Ulnar pCMAP (mV)	6.2 ± 3.2	2.9 ± 2.5	0.010^*^		
Ulnar MNCV (m/s)	48 ± 8.4	41.9 ± 10.2	0.132		
Peroneal DML (ms)	5.6 ± 3.2	5.1 ± 3.8	0.742		
Peroneal dCMAP (mV)	4.1 ± 2.5	1.4 ± 2.1	0.010^*^		
Peroneal pCMAP (mV)	3.7 ± 2.5	1.2 ± 2	0.012^*^		
Peroneal MNCV (m/s)	39.9 ± 5.5	24 ± 16.2	0.009^*^		
Tibial DML (ms)	5.2 ± 2.9	5.4 ± 2.1	0.882		
Tibial dCMAP (mV)	9.6 ± 6.7	2.6 ± 1.8	0.003^*^		
Tibial pCMAP (mV)	7.7 ± 5.5	1.8 ± 1.7	0.002^*^		
Tibial MNCV (m/s)	38.2 ± 7.2	23.1 ± 12.8	0.001^*^		

(B) Sensory nerve conduction study					
	Good (*n* = 13)	Poor (*n* = 11)	*p* value		
Median onset DL (ms)	2.6 ± 2.4	1.6 ± 1.6	0.252		
Median dSNAP (µV)	23.5 ± 20	10.2 ± 11.7	0.066		
Median SNCV (m/s)	42.2 ± 29.6	24.3 ± 28.1	0.147		
Ulnar onset DL (ms)	1.9 ± 0.9	1.8 ± 1.3	0.881		
Ulnar dSNAP (µV)	24.5 ± 21.3	14.4 ± 13.5	0.174		
Ulnar SNCV (m/s)	46.3 ± 26.8	34.2 ± 27.4	0.287		
Sural onset DL (ms)	2.5 ± 1.2	1.4 ± 1.6	0.070		
Sural dSNAP (µV)	13.9 ± 7.7	9.3 ± 12.7	0.286		

(C) F wave, H reflex, needle electromyography					
	Nerve	Good (*n* = 13)	Poor (*n* = 11)	*p* value	OR (95% CI)
Abnormal F wave, N (%)	Median	5 (38.5)	7 (63.6)	0.219	2.8 (0.5–14.7)
	Ulnar	6 (46.2)	4 (36.4)	0.628	0.7 (0.1–3.4)
	Peroneal	8 (61.5)	10 (90.9)	0.098	6.3 (0.6–64.9)
	Tibial	2 (15.4)	7 (63.6)	0.015^*^	9.6 (1.4–67.2)
Abnormal H reflex, N (%)	8 (61.5)	10 (90.9)	0.166	6.3 (0.6–64.9)	
Fibrillation, N (%)	2 (15.4)	4 (36.4)	0.635	2.3 (0.3–16.5)	

(D) Demyelination feature					
	Good (*n* = 13)	Poor (*n* = 11)	*p* value	OR (95% CI)	
Conduction block^a^	4 (30.8)	9 (81.8)	0.012*	10.1 (1.5–70.0)	
Temporal dispersion^b^	0 (0)	5 (45.5)	0.006*	—	
Sural sparing^c^	2 (15.4)	2 (18.2)	11	1.2 (0.1–10.5)	

(E) Distal CMAP threshold					
	Good (*n* = 13)	Poor (*n* = 11)	*p* value	OR (95% CI)	
Median (<4 mV), N (%)	3 (25)	8 (72.7)	0.022^*^	8 (1.2–51.5)	
Ulnar (<6 mV), N (%)	3 (25)	10 (90.9)	0.003^*^	30 (2.6–342.7)	
Peroneal (<2 mV), N (%)	3 (23.1)	9 (81.8)	0.004^*^	15 (2.0–111.2)	
Tibial (<4 mV), N (%)	3 (23.1)	9 (81.8)	0.004^*^	15 (2.0–111.2)	

Abbreviations: 95% CI, 95% confidence interval; dCMAP, distal compound motor action potential; DL, distal latency; DML, distal motor latency; dSNAP, distal sensory nerve action potential; MNCV, motor nerve conduction velocity; OR, odds ratio; pCMAP, proximal compound motor action potential; SNCV, sensory nerve conduction velocity. * Statistical significance at a p‐value <0.05.

^a^ Conduction block, indicated by >20% reduction in CMAP area or amplitude during proximal versus distal nerve stimulation, except in the tibial nerve, for which >50% reduction must be observed in proximal CMAP area or amplitude.

^b^ Temporal dispersion, indicated by >20% increase in the duration of proximal CMAP with a reduction in proximal CMAP amplitude (compared with distal CMAP amplitude).

^c^ Sural sparing was defined as absent or abnormal median sensory nerve action potential (SNAP) amplitude or absent/abnormal ulnar SNAP amplitude with a normal sural SNAP amplitude.

### 3.5 Comparison of Electromyographic Features in the AIDP Subgroup

Based on clinical manifestations and EMG studies, we classified GBS into three types: demyelination, axonal, and Millar‐Fisher. There were 19 patients with acute inflammatory demyelinating polyradiculoneuropathy (AIDP), three with axonal type, and two with Millar–Fisher type (Table 1). Due to the distinct pathophysiology, electrodiagnostic patterns, treatment responses, and outcomes of Miller–Fisher syndrome (MFS) compared to AIDP, we specifically excluded patients with MFS from the analysis.

In the AIDP patients, there are 10 patients with good outcomes and nine patients with poor outcomes. The differences in clinical and electromyographic features between the good and poor outcomes of the AIDP subgroup were similar to those of all GBS patients. The poor outcome AIDP patients were older (61.6 ± 16.5 vs. 38.8 ± 19.7, *p =* 0.015), having more abrupt onset and early progression leading to early medical help‐seeking (onset to hospital interval 1.9 ± 1.8 vs. 9.4 ± 8.7 days, *p =* 0.024), longer hospital stay (54.8 ± 20.5 vs. 13.7 ± 11.6 days, *p* < 0.001), having weaker muscle power with lower MRC sum scores at the first week and fourth week (30.2 ± 16.4 vs. 54.5 ± 5.6, *p =* 0.002; 34.9 ± 16.6 vs. 54.5 ± 5.9, *p =* 0.002), and higher GBS disability scale scores at the first week and fourth week (4.1 ± 0.8 vs. 1.6 ± 1.0, *p* < 0.001; 3.8 ± 0.7 vs. 1.2 ± 0.4, *p* < 0.001) than the good outcome patients. Additionally, the mEGOS outcome scores at the first week (7.9 ± 2.3 vs. 1.5 ± 1.9, *p* < 0.001) and the EGRIS scores at admission (2.8 ± 0.8 vs. 1.5 ± 1.3, *p =* 0.020) also distinguish those AIDP patients with poor and good outcomes (Table A1).

In NCS and electromyographic studies, motor nerve studies were found to be the most effective in distinguishing poor and good outcomes for AIDP patients. Median and tibial CMAP and MNCV were significantly different between poor and good outcome AIDP (median distal CMAP [2.5 ± 2.0 vs. 5.9 ± 2.6, *p =* 0.006], proximal CMAP [1.9 ± 1.6 vs. 5.3 ± 2.5, *p =* 0.004], and MNCV [40.9 ± 9.9 vs. 49.8 ± 5.0, *p =* 0.028]; tibial distal CMAP [2.7 ± 1.8 vs. 9.5 ± 7.4, *p =* 0.014], proximal CMAP [1.9 ± 1.8 vs. 7.8 ± 6.1, *p =* 0.006], and MNCV [22.3 ± 14.2 vs. 38.6 ± 6.6, *p =* 0.005]). Additionally, abnormal F wave in the tibial nerve (77.8% vs. 20.0%, *p =* 0.023) and temporal dispersion in electromyography (55.6% vs. 0%, *p =* 0.011) were also discriminative to poor and good outcomes of the AIDP subgroup (Table A2).

## 4 Discussion

Several factors have been identified as potential early indicators of poor outcomes in patients with GBS (Rajabally and Uncini 2012). In accordance with previous studies, demographics, especially age, are an indicator of GBS outcome (Inés González‐Suárez et al. 2013; Park and Kim 2016). Besides the known demographic predictors, we also identify electrodiagnostic and clinical data as potential indicators of clinical outcomes in this study.

### 4.1 Correlations Between Electrodiagnostic Features and Poor Outcomes

Certain electrodiagnostic features can serve as early indicators of poor outcomes. These features include low CMAP at both distal and proximal sites, reduced motor nerve conduction velocity, increased F‐wave abnormalities, and conduction block and temporal dispersion. First, a mean distal CMAP amplitude of <20% of the lower limit of normal has been reported to be the most important predictor of poor outcomes; a combination of distal CMAP and ventilatory status influence patients’ clinical outcomes over a 4‐week period (Fletcher et al. 2000). In general, the causes of reduction of CMAP could be axonal injury, distally located demyelination, neuromuscular transmission block, and muscle fiber loss. Notably, the findings of the present study highlighted the importance of both distal and proximal CMAP of all major nerves in differentiating the GBS patients with good and poor outcomes. In the subgroup of patients with AIDP, the distal and proximal CMAP of the median and tibial nerves were selectively important in identifying those with poor outcomes.

A reduction in nerve conduction velocity, a demyelinating feature in GBS, is also associated with poor outcomes. Although demyelination in GBS may be reversible, decreases in motor conduction velocity represent a more severe disease. In developing GBS, infiltration of lymphocytes, especially macrophages, complement activation, and vesiculation of the myelin sheath are observed in demyelination; these pathological changes decrease the electric conduction of nerves (Goodfellow and Willison 2016). MNCV is usually decreased in both proximal and distal segments, indicating a diffuse nerve injury in GBS (King and Ashby 1976). In the present study, MNCV of median, peroneal, and tibial nerve differentiated GBS patients with good and poor outcomes. Therefore, slow MNCV is a common electrodiagnostic finding of GBS (Clouston et al. 1994; Ropper, Wijdicks, and Shahani 1990) and also a crucial indicator of GBS outcome.

F response abnormalities indicate proximal demyelination and constitute a demyelinating feature. A previous study showed that abnormal F responses along with abnormal deep tendon reflexes are poor prognostic factors (Lee et al. 2016). The present study further suggested the tibial F responses specifically differentiate GBS patients with good from bad outcomes.

Conduction block and temporal dispersion are traditionally viewed as the two other representative features of demyelinating features. The present study showed that conduction block and temporal dispersion were associated with poor outcomes. A previous study showed that conduction block in the common peroneal nerve was associated with an elevated risk of respiratory failure; in the aforementioned study, a more severe blockage was observed in patients who required ventilation than those who did not require ventilation (Durand et al. 2006). However, some experts suggest that conduction block indicates axonal pathology and is reversible; in these cases, conduction block can occur alone without temporal dispersion (Durand et al. 2006; Uncini and Kuwabara 2018). Conduction block seemed to be different indicators for demyelinating and axonal types of GBS; conduction block indicated poorer outcomes in patients with AIDP type than those with acute motor axonal neuropathy (Niu et al. 2018).

In summary, the initial NCS and electromyography features can differentiate GBS patients with good and poor outcomes. Therefore, comprehensive electrodiagnostic evaluation at the initial encounter of GBS patients and well‐interpretation of the results is valuable to identify patients at a risk of poor outcomes and facilitate timely medical intervention.

### 4.2 Key Clinical Features of Poor Outcomes

Knowledge regarding the pattern of GBS onset is crucial for understanding short‐term outcomes. An aggressive disease onset, characterized by a short onset‐to‐hospitalization period (<10 days), has been associated with a poor prognosis, possibly due to the rapid destruction of peripheral nerves. When nerve damage occurs more rapidly in the early stage of GBS, more time may be needed for functional recovery. A time‐to‐treatment of <7 days may indicate an increased risk of mechanical ventilation requirement (Rajabally and Uncini 2012).

Disease severity at admission and a week after admission are potential indicators of short‐term outcomes. In our study, the initial MRC sum score and GBS Disability Scale score at admission could not effectively differentiate between the poor and good outcome groups. Nonetheless, when measured a week after admission, these scores could effectively predict clinical outcomes. Unlike our study, a study revealed that a low MRC score at admission is strongly associated with a poor short‐term prognosis (Wen et al. 2021).

The duration of hospitalization tends to be longer for patients with poorer outcomes. This is likely attributable to the need for intensive care, rehabilitation, and additional medical care. However, the investment of medical resources could be rewarding for GBS patients. For example, persistent rehabilitation can still confer recovery benefits, extending up to 12 months (Shang et al. 2021).

Mechanical ventilation extends the duration of hospitalization. Approximately 25%–30% of all patients with GBS require mechanical ventilation for respiratory failure (Fourrier et al. 2011; Lee et al. 2016; Wen et al. 2021). In our study, the proportion of patients requiring mechanical ventilation (17%) was slightly lower than that reported in the literature; however, all patients who required mechanical ventilation exhibited poor clinical outcomes. Therefore, the involvement of respiratory muscles and progression to respiratory failure worsen clinical outcomes in patients with GBS.

### 4.3 Identification of High‐Risk Patients by Using Outcome Scoring Systems

Our results indicated that the time‐to‐treatment period plays a key role in determining the EGRIS, a pivotal predictor of clinical outcomes. The MRC sum score at admission and facial and bulbar involvement did differ significantly in predicting clinical outcomes (*p =* 0.128 and *p =* 0.562, respectively). However, a week after admission, the MRC sum score emerged as a valuable predictor of clinical outcomes (*p* < 0.001).

The mEGOS measured at admission and that measured a week after admission is crucial for outcome prediction. Patient age and the MRC sum score on Day 7 are the predominant factors that influence clinical outcomes. Unlike these factors, the MRC sum score at admission and diarrhea did not emerge as significant predictors of clinical outcomes. Similarly, a study using the mEGOS reported that diarrhea was not identified to be a significant predictor of clinical outcomes (Walgaard et al. 2011). Higher mEGOS and EGRIS at admission, particularly scores of >3, indicate higher risks of poor outcomes. Another study indicated that an EGRIS of <5 is associated with improved functional recovery (Kumar et al. 2021).

In summary, key factors influencing clinical outcomes include patient age, time‐to‐treatment period, and MRC sum score on day 7. By contrast, the MRC sum score at admission, facial and bulbar involvement, and diarrhea are weak predictors of clinical outcomes. These insights may guide the refinement of these scoring systems into a simpler formula for future use.

### 4.4 The Outcome Indicators in the AIDP Subgroup

Patients with AIDP type show similar results to those of all GBS patients. However, the clinical feature of a rapidly progressive onset pattern is specifically important. The MCNV, proximal and distal CMAP of the median and tibial nerves, the abnormal tibial F responses, and temporal dispersion are the selected features to distinguish AIDP patients with poor from good outcomes (see Tables A1 and A2). Therefore, these clinical and electrodiagnostic features should be taken into consideration when evaluating AIDP patients. Our study found that over 80% of cases had AIDP, and less than 20% with axonal phenotype. Its conflict to other studies may result from selection bias, and very early studies may misclassify patients with axonal injury as having only demyelinating injury.

### 4.5 Limitations

This study has several limitations. First, despite our efforts to include a substantial number of samples, the sample size was relatively small; this restricted the statistical methods and reduced the analytical power, preventing us from establishing a robust outcome prediction model or refining the current tools. Second, patients with clinically suspected GBS but lacked electrodiagnostic testing were excluded. This represents a significant source of bias. Third, our patients received NCS examinations all in 3 weeks and may lack the sensitivity to accurately identify axonal injury. In addition, although long‐term follow‐up, such as 12‐month outcomes, would be valuable to expand the utilities of the initial electrodiagnostic features in outcome prediction, this was beyond the scope of our current dataset and represents a limitation of this study. Future study design for long‐term follow‐up would help determine the value of the initial electrodiagnostic feature for long‐term outcomes.

As this study was conducted retrospectively, the time between the onset of GBS and the electromyography study varied among patients. This is because the different stages of GBS may have an impact on the results of the EMG study. In addition, some patients received IVIG or plasmapheresis treatment prior to the EMG study, which may have also influenced the results of the study. In the future, we will collect additional GBS data to effectively integrate electromyography features into outcome measurement scales and develop an efficient model for predicting clinical outcomes in patients with GBS.

## 5 Conclusion

In this study, we identified risk factors for poor outcomes in patients with GBS. These factors include older age, a brief time‐to‐treatment period, high mEGOS and EGRIS outcome prediction scores at admission, and specific electrophysiological features of the initial NCS and electromyographic studies such as reduced distal CMAP and proximal CMAP and slow MNCV all major nerves, tibial F‐wave abnormalities, and the presence of conduction block and temporal dispersion. For the AIDP subgroup, the general distinguishing features of poor and good outcomes were similar with all GBS patients. However, median and tibial motor conduction features, including lower distal and proximal CAMP and slower MNCV, were particularly important. By using demographics, initial clinical patterns, and detailed evaluations of electrodiagnostic studies, we can identify those GBS patients at risk of poor outcomes and, therefore, draw medical attention and medical source investigation on these patients.

## Author Contributions

**Yi‐Hsiang Chen**: conceptualization, methodology, data curation, formal analysis, writing–original draft, visualization. **Chia‐Lun Wu**: conceptualization, methodology, validation, supervision, project administration, writing–review and editing, resources. **Wei‐Chieh Weng**: Investigation, supervision, project administration, resources. **Yi‐Chia Wei**: Conceptualization, data curation, formal analysis, supervision, investigation, methodology, validation, project administration, writing–review and editing, resources.

### Peer Review

The peer review history for this article is available at https://publons.com/publon/10.1002/brb3.70257.

## Data Availability

The data that support the findings of this study are available on request from the corresponding author. The data are not publicly available due to privacy or ethical restrictions.

## APPENDIX A

### A.1

**TABLE A1:** Clinical features of the AIDP subgroup.

		Good (N = 10)	Poor (N = 9)	*p* value	OR (95% CI)
Demographics					
Age (years)		38.8 ± 19.7	61.6 ± 16.5	0.015*	
Sex male N (%)		2(20)	1(11.1)	1	0.5 (0–6.7)
Clinical features					
Preceding infection, N (%)	URI	1(10)	3(33.3)	0.303	4.5 (0.4–54.2)
	Diarrhea	1(10)	2(22.2)	0.582	2.6 (0.2–34.5)
Facial or bulbar involvement, N (%)		3(30)	2(22.2)	1	0.7 (0.1–5.3)
Onset to hospital (days)		9.4 ± 8.7	1.9 ± 1.8	0.024*	
Onset to electromyography (days)		14.1 ± 8.1	7.0 ± 6.3	0.049*	
Onset to treatment (days)		14.1 ± 8.6	4.7 ± 1.6	0.011*	
Length of hospital stay (days)		13.7 ± 11.6	54.8 ± 20.5	<0.001*	
Need mechanical ventilator, N (%)		0(0)	3(33.3)	0.087	—
Treatment, N (%)	DFPP	8(80)	9(100)	0.474	—
	IVIG	2(20)	0(0)	0.474	—
Laboratory data					
CSF protein		115.1 ± 58.5	126.4 ± 104.6	0.780	
CSF white blood cell count		2.7 ± 3.3	3.0 ± 4.9	0.867	
Severity of disease					
MRC sum scores at admission		52.4 ± 5.6	50.6 ± 9.4	0.618	
MRC sum scores at first week		54.5 ± 5.9	30.2 ± 16.4	0.002*	
MRC sum scores at fourth week		59.4 ± 1.9	34.9 ± 16.6	0.002*	
GBS disability scale at admission		1.9 ± 0.6	2.4 ± 1.6	0.354	
GBS disability scale at first week		1.6 ± 1	4.1 ± 0.8	<0.001*	
GBS disability scale at fourth week		1.2 ± 0.4	3.8 ± 0.7	<0.001*	
Outcome scores					
mEGOS at admission		1.5 ± 1.3	2.9 ± 2	0.088	
mEGOS at first week		1.5 ± 1.9	7.9 ± 2.3	<0.001*	
EGRIS at admission		1.5 ± 1.3	2.8 ± 0.8	0.020*	

Abbreviations: CSF, cerebrospinal fluid; DFPP, double filtration plasmapheresis; EGRIS, Erasmus GBS Respiratory Insufficiency Score; IVIG, intravenous immunoglobulin; mEGOS, modified Erasmus GBS Outcome Score; MRC, Medical Research Council; URI, upper respiratory tract infection. *Statistical significance at a p‐value <0.05.

**TABLE A2:** Nerve conduction studies and electromyography features of the AIDP subgroup.

MNCV					
	**Good (n=10)**	**Poor (n=9)**	**p**		
**Median DML (ms)**	7.3±5.4	9.3±6.2	.470		
**Median dCMAP (mV)**	5.9±2.6	2.5±2.0	.006*		
**Median pCMAP (mV)**	5.3±2.5	1.9±1.6	.004*		
**Median MNCV (m/s)**	49.8±5.0	40.9±9.9	.028*		
**Ulnar DML (ms)**	4.0±2.8	4.2±1.8	.822		
**Ulnar dCMAP (mV)**	8.0±6.7	4.0±2.6	.110		
**Ulnar pCMAP (mV)**	5.0±2.6	2.9±2.6	.110		
**Ulnar MNCV (m/s)**	47.3±9.3	41.9±11.4	.287		
**Peroneal DML (ms)**	6±3.5	5.7±3.8	.837		
**Peroneal dCMAP (mV)**	4.1±2.8	1.6±2.3	.053		
**Peroneal pCMAP (mV)**	3.7±2.8	1.4±2.1	.061		
**Peroneal MNCV (m/s)**	39.4±6.0	26±15.7	.023*		
**Tibial DML (ms)**	5.6±3.2	5.5±2.1	.883		
**Tibial dCMAP (mV)**	9.5±7.4	2.7±1.8	.018*		
**Tibial pCMAP (mV)**	7.8±6.1	1.9±1.8	.014*		
**Tibial MNCV (m/s)**	38.6±6.6	22.3±14.2	.005*		

Demyelination feature					
	**Good (n=10)**	**Poor (n=9)**	**P**	**OR (95%CI)**	
**Conduction block**	4(40)	7(77.8)	.170	5.3(0.7‐39.5)	
**Temporal dispersion**	0(0)	5(55.6)	.011*	—	
**Sural sparing**	2(20)	2(22.2)	1	1.1(0.1‐10.4)	

SNCV					
	**Good (n=10)**	**Poor (n=9)**	**P**		
**Median onset DL (ms)**	2.5±2.7	1.5±1.6	.364		
**Median dSNAP (µV)**	26.6±22	9.6±11.2	.052		
**Median SNCV (m/s)**	37.4±32.3	24.1±28.7	.357		
**Ulnar onset DL (ms)**	1.7±1.0	1.9±1.3	.750		
**Ulnar dSNAP (µV)**	27.6±23.5	14.1±13	.139		
**Ulnar SNCV (m/s)**	43.2±29.9	36.4±27.6	.619		
**Sural onset DL (ms)**	2.4±1.4	1.7±1.6	.264		
**Sural dSNAP (µV)**	13.9±8.7	11.4±13.2	.630		

F wave, H reflex, needle electromyography					
	**Nerve**	**Good (n=10)**	**Poor (n=9)**	**P**	**OR (95%CI)**
**Abnormal F wave, N (%)**	**Median**	4(40.0)	7(77.8)	.170	5.3(0.7‐39.5)
	**Ulnar**	5(50.0)	4(44.4)	1	0.8(0.1‐4.9)
	**Peroneal**	7(70.0)	8(88.9)	.582	3.4(0.3‐40.9_
	**Tibial**	2(20.0)	7(77.8)	.023	14.0(1.5‐127.2)
**Abnormal H reflex, N (%)**		6(60.0)	8(88.9)	.303	5.3(0.5‐60.8)
**Fibrillation, N (%)**		1(14.3)	2(22.2)	1	1.7(0.1‐23.9)

Distal CMAP threshold					
	**Good (n=10)**	**Poor (n=9)**	**P**	**OR (95%CI)**	
**Median (< 4mV), N (%)**	3(33.3)	7(77.8)	.153	7.0(0.9‐56.9)	
**Ulnar (< 6mV), N (%)**	3(33.3)	8(88.9)	.050	16.0(1.3‐194.6)	
**Peroneal (< 2mV), N (%)**	3(30.0)	7(77.8)	.070	8.2(1‐64.9)	
Tibial (< 4mV), N (%)	3(30.0)	7(77.8)	.070	8.2(1‐64.9)	

Abbreviations: DL, distal latency; DML, distal motor latency; dCMAP, distal compound motor action potential; dSNAP, distal sensory nerve action potential; OR, odds ratio; 95% CI, 95% confidence interval; MNCV, motor nerve conduction velocity; pCMAP, proximal compound motor action potential; SNCV, sensory nerve conduction velocity. *Statistical significance at a p‐value <0.05.
